# Inhibition of Ceramide Glycosylation Enhances Cisplatin Sensitivity in Cholangiocarcinoma by Limiting the Activation of the ERK Signaling Pathway

**DOI:** 10.3390/life12030351

**Published:** 2022-02-28

**Authors:** Piyasiri Chueakwon, Peeranat Jatooratthawichot, Krajang Talabnin, James R. Ketudat Cairns, Chutima Talabnin

**Affiliations:** 1 School of Chemistry, Institute of Science, Suranaree University of Technology, Nakhon Ratchasima 30000, Thailand; p.chueakwon@gmail.com (P.C.); pj.cbsfa@gmail.com (P.J.); cairns@sut.ac.th (J.R.K.C.); 2 School of Pathology, Institute of Medicine, Suranaree University of Technology, Nakhon Ratchasima 30000, Thailand; krajang.t@sut.ac.th

**Keywords:** glucosylceramide synthase, PPMP, cisplatin, sensitivity, cholangiocarcinoma

## Abstract

Cholangiocarcinoma (CCA) is an aggressive tumor of the biliary epithelium with poor survival that shows limited response to conventional chemotherapy. Increased expression of glucosylceramide synthase (GCS) contributes to drug resistance and the progression of various cancers; the expression profiles of GCS (UGCG) and the genes for glucocerebrosidases 1, 2, and 3 (GBA1, GBA2, and GBA3) were therefore studied in CCA. The biological functions of GCS for cell proliferation and cisplatin sensitivity in CCA were explored. GCS expression was higher in CCA tumor tissues than that of GBA1, GBA2, and GBA3. Verification of GCS expression in 29 paired frozen CCA tissues showed that 8 of 29 cases (27.6%) had high GCS expression. The expression of GCS and GBA2 was induced in CCA cell lines following low-dose cisplatin treatment. Suppression of GCS by either palmitoylamino-3-morpholino-1-propanol (PPMP), GCS knockdown or a combination of the two resulted in reduced cell proliferation. These treatments enhanced the effect of cisplatin-induced CCA cell death, increased the expression of apoptotic proteins and reduced phosphorylation of ERK upon cisplatin treatment. Taken together, inhibition of the GCS increased cisplatin-induced CCA apoptosis via the inhibition of the ERK signaling pathway. Thus, targeting GCS might be a strategy for CCA treatment.

## 1. Introduction

Cholangiocarcinoma (CCA) is one of the most common aggressive malignancies of the bile duct epithelium, with an increasing worldwide incidence, particularly in Southeast Asia and Eastern Europe [1]. Late diagnosis, inoperable advanced stage, and resistance to chemotherapy remain major obstacles to CCA treatment [2,3]. Conventional chemotherapeutic agents—including 5-fluorouracil, cisplatin, leucovorin, mitomycin, gemcitabine, and cisplatin—have been used either alone or in combination with a response rate ranging between 20 and 30% [4,5]. The current first-line chemotherapeutic regimen used in CCA treatment is a combination of gemcitabine and cisplatin [6]. However, the effectiveness of this regimen has been modest as the 5-year survival of 564 CCA patients between 1973 and 2004 was 18% [7,8]. A new treatment option for CCA is thus urgently needed.

Cisplatin, a platinum-based drug, is a chemotherapeutic drug widely applied in various types of cancers, including CCA. Cisplatin is usually combined with 5-fluorouracil or gemcitabine to increase the response rate and overall survival of CCA patients [9]. According to its cytotoxic mode of action, cisplatin mainly interacts with DNA, leading to DNA adduct-induced DNA damage and subsequent induction of apoptosis through the activation of the p53 and MAPK pathways [10]. Numerous studies have demonstrated that cisplatin-induced cell apoptosis is involved in an imbalance in sphingolipid levels. Ceramide (Cer) is a second messenger in sphingolipid metabolism which can function as a potent tumor suppressor lipid to mediate cell death by triggering cell growth arrest as an inducer of apoptosis, necroptosis, and mitophagy [11,12]. Induction of ceramide generation has been characterized as another cytotoxic mode of cisplatin leading to ceramide-mediated mitochondrial apoptosis and/or ceramide-mediated death through the Fas pathway [13,14,15].

Ceramide glycosylation is the first step in the biosynthesis of glycosphingolipids, and it has been implicated in the multi-drug resistance of various cancers [16]. Ceramide glycosylation occurs by the action of glucosylceramide synthase (GCS), which is encoded by the GCS (UGCG) gene. GCS is a transmembrane protein at the Golgi surface, converting Cer to a precursor of most of the complex glycosphingolipids (GSLs), glucosylceramide (GlcCer), by transferring the glucosyl residue from UDP-glucose to the hydroxyl group at C1 of ceramide [17]. Notwithstanding, the level of GlcCer is regulated by hydrolysis, either in the lysosome by glucocerebrosidase 1 (GBA1) or on the cytoplasmic side of the ER and Golgi membranes by glucocerebrosidase 2 (GBA2) [18]. Up-regulation of GCS expression has been reported in multiple cancer types, including cervical [19], breast [12], lung [20], colon [19,21], and hepatocellular carcinoma [22]. Its expression is correlated with various aspects of cancer progression, including chemotherapeutic drug resistance, cell growth, and stemness properties through the activation of the AKT/ERK and c-Src/β-catenin signaling pathways [12]. Currently, several lines of evidence have confirmed that GCS plays a major role in resistance to a variety of chemotherapeutic agents through the up-regulation of multi-drug resistant genes (MDR1) [12]. Therefore, the targeting of GCS is considered a means of overcoming cancer progression and drug resistance in various cancers; however, the GCS expression level in CCA and its role in CCA chemoresistance remains unknown. In the current study, the expression level of GCS was determined in CCA tissues and CCA cell lines. The biological effects of GCS on CCA cell growth, cisplatin sensitivity, and its underlying mechanisms were studied.

## 2. Materials and Methods

### 2.1. GEO Database

Gene expression data of CCA were retrieved from the public database—Gene Expression Omnibus (GEO) (https://www.ncbi.nlm.nih.gov/geo/, accessed on 13 April 2020)—through GEO series GSE76297 [23] on 13 April 2020. GEO series GSE76297 contains the expression data from 91 CCA tumors and 92 paired non-tumors. All expression data were log2 transformed.

### 2.2. CCA Tissue Samples and Cell Lines

The twenty-nine frozen CCA tissues and paired adjacent tissues were obtained from the specimen bank of the Cholangiocarcinoma Research Institute, Khon Kaen University. Informed consent was obtained from each patient. The research protocol was reviewed and approved by the Ethics Committee for Human Research of Khon Kaen University (HE521209) and Suranaree University of Technology (EC-57-25).

Two human CCA cell lines (KKU-100 and KKU-213A) were established and authenticated [24,25]. Certificates of analyses were obtained from the Japanese Collection of Research Bioresources Cell Bank. The cells were cultured in DMEM (Thermo Fischer Scientific, Grand Island, NY, USA) supplemented with 1% penicillin-streptomycin (Thermo Fischer Scientific, Grand Island, NY, USA) and 10% FBS (Thermo Fischer Scientific, Grand Island, NY, USA) and maintained in a humidified incubator with 5% CO_2_ at 37 °C. Mycoplasma testing was performed for the cell lines using PCR detection and Hoechst 33258 staining Dye solution (H3569, Thermo Fischer Scientific, Waltham, MA, USA).

### 2.3. Quantitative Real-Time PCR (qPCR)

Total RNA was extracted using Trizol (Thermo Fischer Scientific, USA) according to the manufacturer’s instructions. cDNA was then synthesized using a SensiFAST cDNA Synthesis Kit (Bioline, Memphis, TN, USA). Quantitative real-time PCR was performed to evaluate the gene expression levels of GCS (UGCG) GBA1 and GBA2 using a LightCycler^®^ 480 SYBR Green I Master (Roche Diagnostic, Mannheim, Germany). The primer sequences were: GBA1 primer (forward: 5′-GTT CCA GAA AGT GAA GGG AT-3′ and reverse 5′-TTC TCT GAA GAA GGA ATC GG-3′); human GBA2 primer (forward: 5′-CCA CTA CAG GCG GTA TAC AA-3′ and reverse 5′-GAT CTG TCA TCC AAT ACC GG-3′); GCS (UGCG) primer (forward: 5′-TGC TCA GTA CAT TGC CGA AGA-3′ and reverse 5′-TGG ACA TTG CAA ACC TCC AA-3′); c-Myc primer (forward: 5′-CTG CTG TGG ACC CTA CTG-3′ and reverse: 5′-AAC TGC GTC TCT GCC AGG AC-3′); CCND1 primer (forward: 5′-CCA CTT GAG CTT GTT CAC CA-3′ and reverse 5′-AAC TAC CTG GAC CGC TTC CT-3′); CDK4 primer (forward: 5′-GTC GGC TTC AGA GTT TCC AC-3′ and reverse 5′-TGC AGT CCA CAT ATG CAA CA-3′); and CCNB1 primer (forward: 5′-GAC AAC TTG AGG AAG AGC AAG C-3′ and reverse 5′-ATG GTC TCC TGC AAC AAC CT-3′). β-actin was used as an internal control (forward: 5′-GAT CAG CAA GCA GGA GTA TGA CG-3′ and reverse 5′-AAG GGT GTA ACG CAA CTA AGT CAT AG-3′). Gene amplification was performed for 40 cycles of denaturation at 95 °C for 10 s, annealing at 58 °C for 10 s, and extension at 72 °C for 10 s. The melting curve (1 cycle) was at 95 °C for 10 s and 65°C for 1 min. Annealing at 58 °C was used for all primers. The relative gene expression levels were normalized with an internal control (β-actin) calculated by 2^−ΔΔCT^ [26].

### 2.4. Protein Collection and Immunoblotting

CCA cells were lysed in lysis buffer: 150 mM NaCl; 50 mM Tris-HCl pH 7.4; 1% Sodium deoxycholate; 0.1% SDS; and 1X Protease inhibitor cocktail tablets (Roche Diagnostic, Mannheim, Germany) to obtain whole cell lysates. The protein concentration of all samples was determined with the Pierce BCA Protein Assay Kit (Thermo Scientific, Rockford, IL, USA). Equal amounts of proteins (30 µg/lane) were resolved by SDS-PAGE (SDS-polyacrylamide gel electrophoresis) using 12% polyacrylamide gel. The proteins were then transferred onto a nitrocellulose membrane (GE Healthcare, Buckinghamshire, UK). The membrane was incubated in 5% skim milk PBST at room temperature for blocking non-specific proteins. Primary antibodies were then probed at 4 °C, overnight, including GCS (ab124296, Abcam, Cambridge, UK), PARP1 (13371-1), BAX (50599-2), BCL-2 (12789-1) (Proteintech, Rosemont, IL, USA), caspase-3 (#9662), cleaved caspase-3 (c-caspase 3) (#9661), AKT (#4685), phosphorylated AKT (pAKT) (#4060), ERK1/2 (#4695), phosphorylated ERK1/2 (pERK) (#9101) (Cell signaling, USA), and β-actin (sc-47778, Santa Cruz Biotechnology, USA). All antibodies were diluted to 1:1000–1:5000. The membrane was then washed with PBST and incubated with horseradish peroxide-conjugated secondary antibodies (1:2000, GE Healthcare, Buckinghamshire, UK). Immunodetection by chemiluminescent HRP substrate was used to visualize the target protein signals (GE Healthcare, Buckinghamshire, UK). Image J software (version 1.53a; National Institutes of Health, Bethesda, MD, USA) was used to determine the density of each target protein, then the relative intensity of the protein band was transformed into a quantitative value. The ratio of each protein band intensity to that of β-actin was calculated for normalization.

### 2.5. Inhibition of GCS by Specific-siRNA

CCA cell lines were plated at 3.5 × 105 cells in 6-well plates. After overnight culture, the cells were transfected with either 10 µM siCTRL (sc-37007, Santa Cruz Biotechnology, Santa Cruz, CA, USA) or 10 µM siGCS (sc-45404, Santa Cruz Biotechnology, Santa Cruz, CA, USA), using DharmaFECT transfection reagents (Horizon, Ireland) in OptiMEM (Thermo Fischer Scientific, Grand Island, NY, USA) for 24 and 48 h. The transfection procedure was performed according to the recommendations of the manufacturer.

### 2.6. Sulforhodamine B (SRB) Assay

Cell viability was assessed by the SRB assay. First, the cells were fixed in 10% (*w*/*v*) tricarboxylic acid (TCA) at 4 °C overnight. After washing, the cells were then stained with SRB dye for 30 min. Next, the residual dye was washed out with 1% (*v*/*v*) acetic acid. The dye-binding proteins were then dissolved in 10 mM Tris base. The absorbance at 564 nm was measured in a microplate reader (Bio-Rad Laboratories, Inc., Hercules, CA, USA) to determine cell viability.

### 2.7. Cell Proliferation

CCA cells were seeded at 5 × 103 cells per well in 96-well plates at 37 °C. After 24 h seeding, the cells were then exposed with or without 10 µM d,l-threo-1-Phenyl-2-palmitoylamino-3-morpholino-1-propanol hydrochloride (PPMP, sc-205655, Santa Cruz Biotechnology, Santa Cruz, CA, USA) and continuously incubated at 37 °C for 24 and 48 h. The cell viability was assessed using the SRB assay. For GCS silencing, the cells were treated with siCTRL or siGCS in 6-well plates. After siRNA transfection, the cells were seeded into 96-well plates overnight, and viable cells were determined using the SRB assay at 24 and 48 h.

### 2.8. Drug Treatment

In order to evaluate the cytotoxicity effect of cisplatin in CCA cell lines, the cells were seeded at 7 × 103 cells per well in 96-well plates at 37 °C for 24 h. Then the cells were treated with cisplatin at various concentrations (0, 20, 40, and 60 µM) for another 24 and 48 h. After investigating the effect of GCS suppression on cisplatin treatment, the GCS-silencing and control cells were seeded into 96-well plates. After seeding for 24 h, the cells were treated with (a) cisplatin alone at 10 and 20 µM; (b) D,L-threo-1-Phenyl-2-palmitoylamino-3-morpholino-1-propanol hydrochloride (PPMP) alone at 10 µM; and (c) co-treatment of cisplatin and PPMP for 24 and 48 h. Cell viability was determined by the SRB assay. The half-maximal inhibitory concentration (IC50) was calculated with GraphPad Prism 8 (GraphPad Software, San Diego, CA, USA).

### 2.9. Statistical Analysis

The data were analyzed with GraphPad Prism 8 (GraphPad Software, San Diego, CA, USA) and IBM SPSS 22.0 software (IBM Corporation, Armonk, NY, USA). The data were expressed as means ± SEMs of at least three independent experiments. The correlation of gene expression data of tumor versus non-tumor from the GEO database was evaluated using the Mann–Whitney test. A Chi-square test was used to analyze the relationship between the expression of GCS and clinical data. Survival analysis was plotted using a Kaplan–Meier curve, and the log-rank test was used to compare curves. A paired sample *t*-test was used to compare sets of paired data. One- and two-way ANOVA were used to compare the two groups for one and two independent variables, respectively. All statistical analyses were two-sided. *p* < 0.05 was considered statistically significant.

## 3. Results

### 3.1. Glucosylceramide Synthase (GCS) Expression in Cholangiocarcinoma Tissues

**Figure 1:**
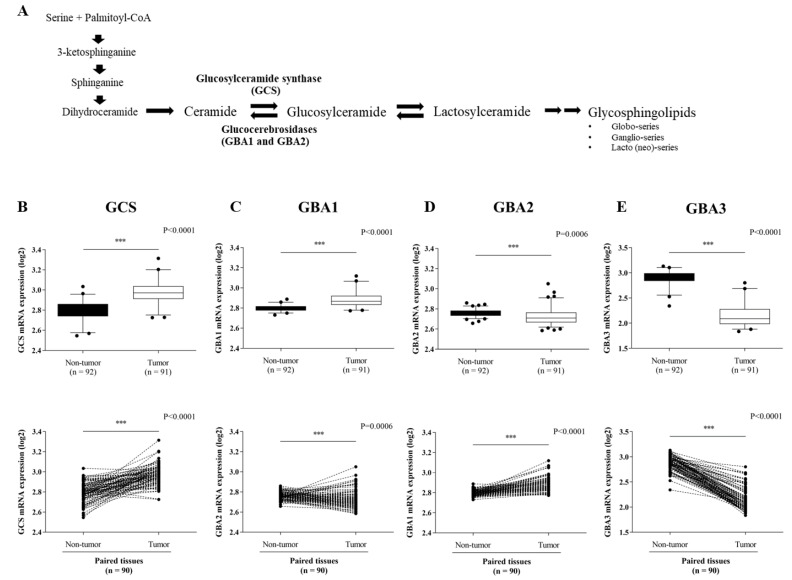
Expression of ceramide-metabolizing enzymes in paired CCA tissues. (**A**) Diagram of the sphingolipids biosynthesis pathways. Expression data were obtained from the GEO database (GSE76297 dataset) for a comparison of: (**B**) *GCS* mRNA expression, (**C**) *GBA1* mRNA expression, (**D***)* *GBA2* mRNA expression and (**E**) *GBA3* mRNA expression. The Mann-Whitney was used to compare the expression between tumor (n = 92) and non-tumor tissues (n = 91) as well as paired tissues. ***, *p* < 0.001 versus non-tumor tissues.

**Figure 2:**
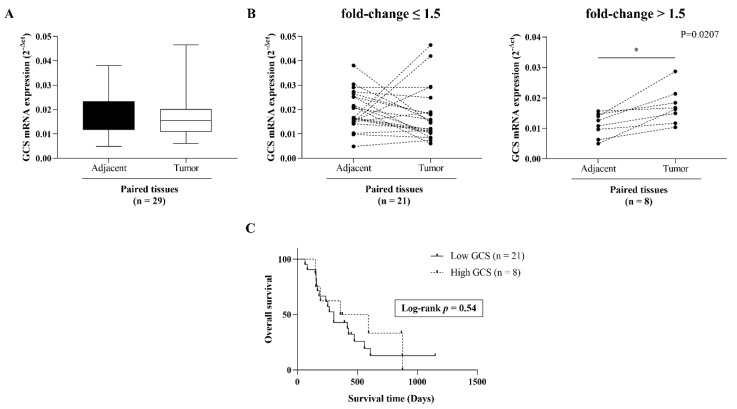
*GCS* expression in 29 paired frozen CCA tissues. (**A**) *GCS* mRNA expression was determined by qPCR. (**B**) Cut-off at 1.5-fold change (tumor/adjacent normal) was used for dichotomized *GCS* mRNA expression in 2 groups. Cut-off value ≤ 1.5-fold change and > 1.5-fold change denoted as low and high *GCS* expression, respectively. (**C**) Kaplan-Meier analysis of overall survival in low and high *GCS* cases. Log Rank test was used for survival analysis. *, *p* < 0.05 versus adjacent normal tissues.

In order to explore the expression level of ceramide-metabolizing enzymes (Figure 1A), including GCS, GBA1, GBA2, and GBA3 in CCA tissues, we first retrieved the expression data of the four genes from the GEO database (GEO Series GSE76297). Then, the differential expression of the four genes was compared between CCA tumor tissues and non-tumor tissues. Both the GCS and the GBA1 expression levels were significantly up-regulated in CCA tumor tissues (Figure 1B,C), whereas the GBA2 and GBA3 were down-regulated compared with matched paired non-tumor CCA tissues (Figure 1D,E). Additionally, the expression level of GCS was higher in the CCA tumor tissues than the other glucocerebrosidase (GBA1, GBA2, and GBA3). GCS expression was further verified in 29 frozen paired CCA tumor tissues and adjacent normal tissues via qPCR (Figure 2A). At a cut-off value set at 1.5-fold difference, high GCS expression was found in 27.6% (8/29 cases) of tumor tissues, whereas in the remaining 72.4% (21/29) of cases, GCS expression was low (Figure 2B). The finding suggests that altered expression of ceramide-metabolizing enzymes occurs in CCA.

In order to address the significance of GCS expression in CCA, we investigated the association between GCS expression and overall survival or clinicopathological features. The Kaplan–Meier analysis revealed no statistically significant correlation between GCS expression and overall survival (log-rank test, *p* = 0.67) (Figure 2C). The univariate analysis demonstrated that there was no statistically significant difference between GCS expression and any clinicopathological variables (including age, sex, tumor stages, node lymphatic invasion, and histological types) (Table 1).

### 3.2. Altered Expression of Ceramide-Metabolizing Enzymes Following Cisplatin Treatment

**Figure 3:**
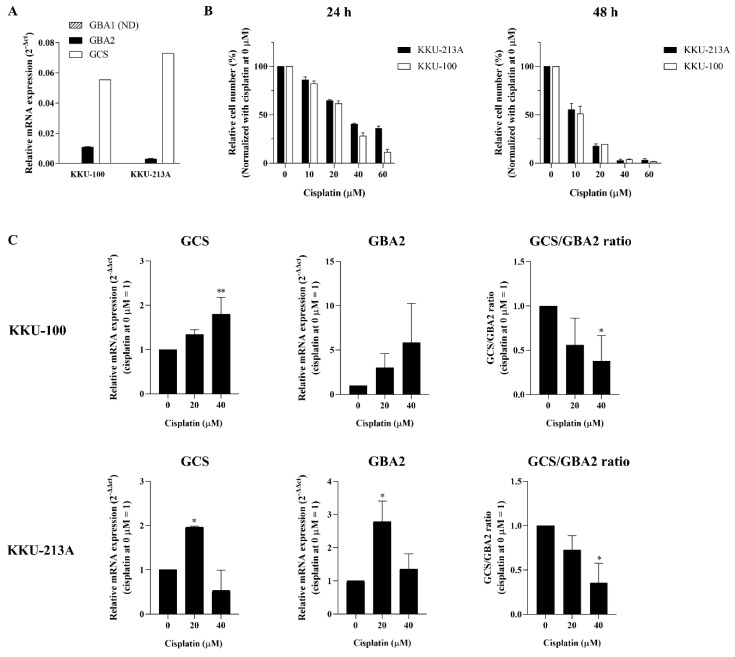
Changes of ceramide-metabolizing enzymes upon cisplatin treatment. (**A**) Respective basal mRNA expression of GCS, GBA1, and GBA2 were determined in two CCA cell lines (KKU-100 and KKU-213A). (**B**) KKU-100 and KKU-213A were treated with cisplatin at 0, 10, 20, 40, and 60 µM for 24 and 48 h. (**C**) Respective mRNA expression of GCS, GBA2, and GCS/GBA2 ratio was determined in KKU-100 and KKU-213A after 24 h of cisplatin treatment. Values are expressed as the mean ± SEM of three independent experiments. * *p* < 0.05; ** *p* < 0.01 versus cisplatin at 0 µM. ND: not detected.

In order to address the role of ceramide glycosylation in CCA, we first determined the basal expression of the three ceramide-metabolizing enzymes (GCS, GBA1, and GBA2) in the two CCA cell lines (KKU-100 and KKU-213A), while basal GBA3 expression was excluded because its specific role is still unclear. A high expression level of GCS was found in both CCA cell lines, whereas the respective expression of GBA1 and GBA2 was low (Figure 3A). We then investigated whether alteration of the ceramide-metabolizing enzymes occurs upon cisplatin treatment of the CCA cell lines. The cytotoxicity effect of cisplatin was tested in the CCA cell lines. The cells were treated with various concentrations of cisplatin at 0, 10, 20, 40, and 60 µM. After 24 and 48 h treatment, cell viability was significantly decreased in a dose- and time-dependent manner. The respective half-maximum inhibitory concentration (IC50) value of cisplatin at 24 and 48 h was 31.17 and 14.72 µM for KKU-100 and 20.98 and 18.82 µM for KKU-213A (Figure 3B). The expression of the three ceramide-metabolizing enzymes was then investigated following cisplatin treatment for 24 h. The gene expression experiments revealed that inducible expression of GCS and GBA2 was observed at 20 µM cisplatin in both KKU-100 and KKU-213A, and the expression of the enzymes declined at 40 µM cisplatin (Figure 3C). The significant induction of GCS and GBA2 expression was most clearly evident in KKU-213A. Additionally, the expression ratio of GCS/GBA2 was decreased in KKU-213A in a dose-dependent manner (Figure 3C). According to the gene expression experiment, GBA1 expression was not detectable in all conditions. Taken together, these findings suggest that the altered expression of GCS and GBA2 would be another anticancer effect of cisplatin in CCA.

### 3.3. Suppression of GCS Reduces CCA Cell Growth

**Figure 4:**
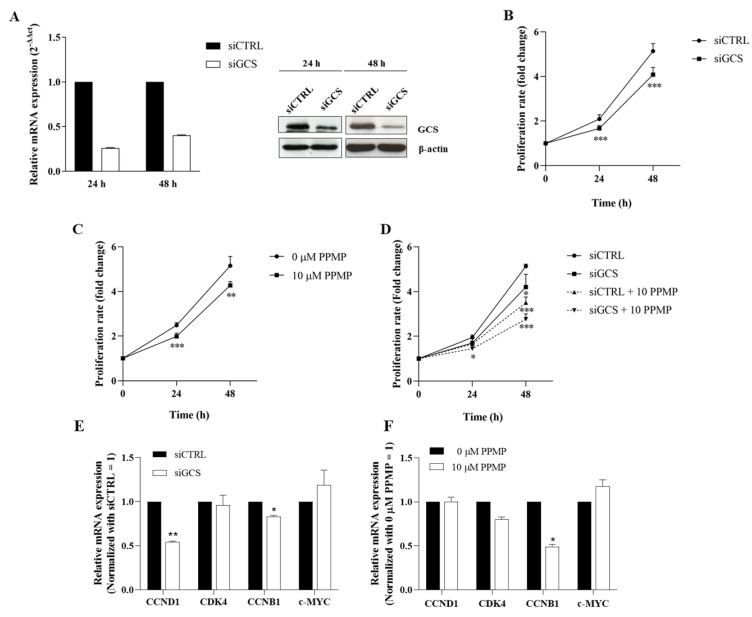
Suppression of GCS reduces CCA cell growth. (**A**) mRNA and protein levels of GCS after suppression by siRNAs for 24 and 48 h. (**B**) Cell proliferation after GCS suppression by siRNAs for 0–48 h, (**C**) PPMP (GCS inhibitor), and (**D**) co-treatment by siRNAs and PPMP. (**E**,**F**) The relative mRNA expression of growth-related genes in siGCS-treated KKU-213A or PPMP-treated KKU-213A were determined using qPCR. Values are expressed as the mean ± SEM of three independent experiments. * *p* < 0.05; ** *p* < 0.01; *** *p* < 0.001 versus siCTRL. The original unedited blot was presented in Figure S1.

In order to evaluate the role of GCS on CCA progression, the effects of GCS knockdown and PPMP (a chemical GCS inhibitor) on CCA cell growth were investigated. Compared to KKU-100, KKU-213A is a highly aggressive CCA cell line with a high growth rate [25]. KKU-213A has the highest basal GCS expression and significant inducible GCS and GBA2 expression following cisplatin treatment. Thus, KKU-213A was selected as the representative CCA cell line for the follow-up experiment. The GCS mRNA and protein levels were significantly decreased in the siGCS-treated KKU-213A compared to the control cells at both 24 h and 48 h (Figure 4A and Figure S1). The SRB assay showed that the GCS knockdown significantly reduced cell proliferation at 24 and 48 h (Figure 4B). A similar observation occurred in the PPMP-treated KKU-213A cells (Figure 4C). The anti-proliferative effect was also clearly seen in the PPMP and GCS knockdown co-treatments. (Figure 4D). Moreover, down-regulation of growth-related genes including CCND1, CCNB1, and CDK4 was observed in both siGCS-treated and PPMP-treated KKU-213 cells (Figure 4E,F). These findings suggest that GCS affects CCA cell growth.

### 3.4. Suppression of GCS Enhances Cisplatin Sensitivity

**Figure 5:**
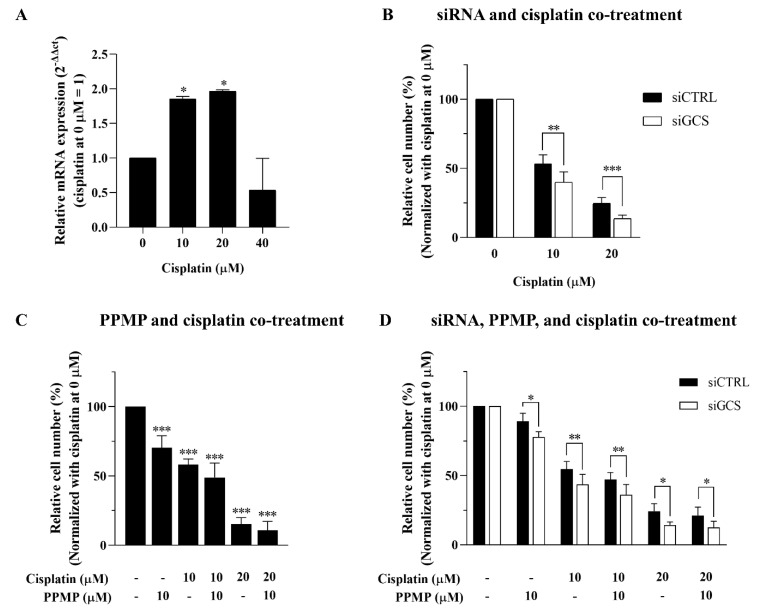
Suppression of GCS enhances cisplatin sensitivity. (**A**) Inducible mRNA expression of GCS following low-dose cisplatin (0, 10, 20, and 40 μM). Cell viability of KKU-213A was determined after (**B**) GCS inhibition by siRNAs for 24 h and treated with cisplatin for 48 h, (**C**) GCS inhibition by PPMP for 24 h and in combination with cisplatin for 48 h, and (**D**) co-treatment of GCS with siRNAs and PPMP for 24 h plus cisplatin for 48 h. Values are expressed as the mean ± SEM of three independent experiments. * *p* < 0.05; ** *p* < 0.01; *** *p* < 0.001 versus cisplatin at 0 µM.

According to Figure 3C, the inducible expression of GCS was seen in the 20 µM cisplatin treatment, and the expression declined following 24 h of 40 µM cisplatin treatment, along with the number of viable cells (Figure 3B). We further investigated whether GCS expression influences the response to cisplatin—especially at low doses (viz., 10 and 20 μM). An inducible expression of GCS was indicated at the 10 and 20 µM cisplatin treatment (Figure 5A). The siGCS-treated KKU-213A cells and the control cells were exposed to 0, 10, and 20 µM of cisplatin. GCS silencing increased cisplatin-induced CCA cell death by reducing viable cells in a dose-dependent manner compared to the control cells (Figure 5B).

Furthermore, GCS suppression using 10 µM PPMP treatment increased the anticancer effect of cisplatin in a dose- and time-dependent manner (Figure 5C). Nevertheless, co-treatment between GCS knockdown, PPMP, and cisplatin showed no further increase in the anticancer effect on CCA cell lines (Figure 5D). This finding suggests that inhibition of GCS using either knockdown or PPMP treatment is sufficient for GCS suppression. Thus, our findings indicate that the inhibition of GCS has a chemosensitizing effect on CCA, such that cisplatin and cisplatin-induced CCA cell death may involve an imbalance in the process of ceramide glycosylation through GCS and GBA2 expression.

### 3.5. Suppression of GCS Promotes Cisplatin-Induced CCA Apoptosis through the Inhibition of the ERK Signaling Pathway

**Figure 6:**
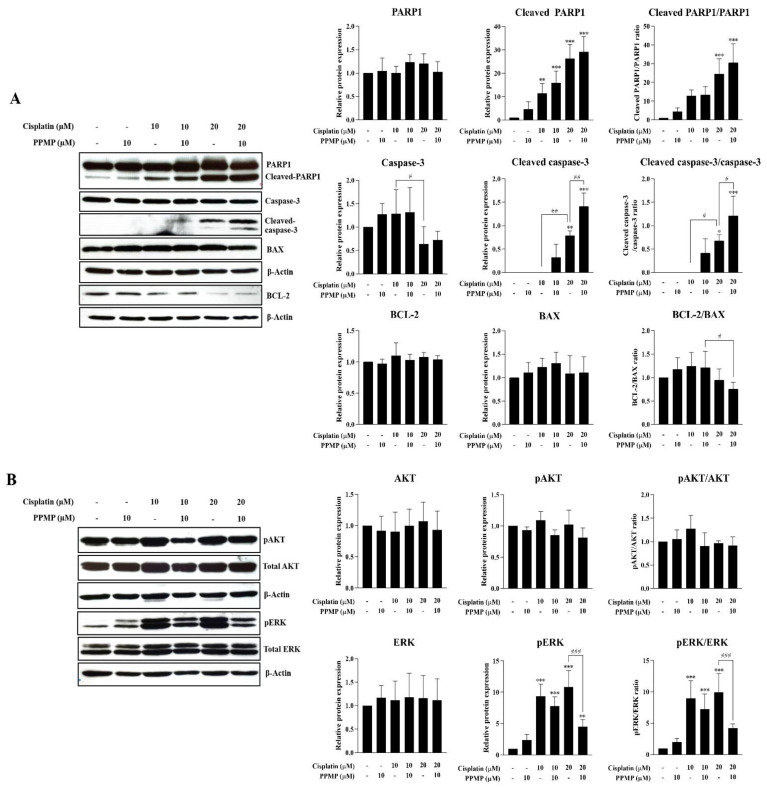
Suppression of GCS induces CCA cell apoptosis via the attenuation of ERK activation. KKU-213A was exposed by PPMP alone (10 µM), cisplatin alone (10 or 20 µM), or the combination of both for 24 h. Protein expression potentially involved (**A**) apoptosis and (**B**) survival signaling pathway observed by Western blot assay. Relative protein expression was measured by Image J software (version 1.53a). Values are expressed as the mean ± SEM of three independent experiments. * *p* < 0.05; ** *p* < 0.01; *** *p* < 0.001 versus cisplatin at 0 µM. ^#^ *p* < 0.05; ^##^ *p* < 0.01; ^###^ *p* < 0.001 versus cisplatin at 10 or 20 µM. The original unedited blot was presented in Figure S2.

In order to elucidate the underlying mechanism of GCS inhibition-increased cisplatin-induced CCA cell death (i.e., in KKU-213A cells), GCS was suppressed by 10 µM PPMP and co-treated with 10 or 20 µM cisplatin. The apoptosis-related markers were then investigated. The immunoblotting assay of KKU-213A confirmed dose-dependent up-regulation of cleaved PARP1 and cleaved caspase-3 and down-regulation of BCL-2 with co-treatments between PPMP and cisplatin. However, a decreased BCL-2 (pro-apoptotic protein)/BAX (apoptotic protein) ratio was observed in the KKU-213A cell line treated with cisplatin alone at 20 µM or co-treated with cisplatin at 20 µM and PPMP at 10 µM. However, none of these apoptotic markers was altered upon PPMP exposure (Figure 6A and Figure S2). Subsequently, the survival pathways (i.e., PI3K/AKT and ERK) were investigated. Interestingly, suppression of GCS significantly reduced the phosphorylation and activation of ERK following cisplatin treatment at 10 and 20 µM, while no activation of AKT was observed after GCS inhibition (Figure 6B and Figure S2). These findings suggest that GCS suppression enhanced cisplatin-induced CCA apoptosis through the attenuation of the ERK signaling pathway.

## 4. Discussion

Ceramide glycosylation eliminates the anticancer effect of ceramide, thereby promoting cancer cell growth and survival [27]. Glucosylceramide synthase is a key enzyme that catalyzes the conversion of ceramide to glucosylceramide, a synthesis precursor to most complex glycosphingolipids (GSLs) [28,29,30]. There is growing evidence that GCS is up-regulated in multiple types of cancer, and its expression is associated with the progression of cancer and drug resistance [22,31,32,33]. According to the GEO database, GCS was overexpressed in CCA tumor tissues and showed higher expression than the two glucosylceramide-degrading enzymes, GBA1 and GBA2. However, verification of GCS mRNA expression in 29 pairs of matched tumor and adjacent normal samples showed only 27.6% (8/29 cases) of tumor tissues having high GCS mRNA expression relative to the adjacent tissue. We demonstrated no significant correlation between GCS mRNA expression, survival, and clinicopathological variables in CCA, possibly because of the small sample used and the low prognostic value of GCS expression in cancer patients. Therefore, the verification of the increased GCS expression at protein levels in large sample size and clinical correlations are required in further investigations. High expression of GCS was, however, confirmed in two CCA cell lines where no GBA1 expression and low GBA2 expression were detected. The results suggest that aberrant expression of ceramide-metabolizing enzymes occurs in CCA. Additionally, high GCS expression in CCA is consistent with the studies on lipidomic measurements in CCA tissues, in which high levels of hexosylceramides and lactosylceramides are observed in CCA tissues, and these levels were associated with short survival of the CCA patients [34,35]. Up-regulation of GCS is implicated in cancer progression and multi-drug resistance, but the role of glucosylceramide breakdown (including GBA1 and GBA2) in cancer is unclear. Expression of GBA1 and GBA2 varies among cancer cell types. For example, down-regulation of GBA1 and GBA2 was found in colon cancer, whereas overexpression of GBA1 was observed in breast cancer [36]. Furthermore, suppression of GBA1 using siRNA induced paclitaxel resistance and the activation of the AKT pathway in three different cancer cell lines, including colon carcinoma, breast adenocarcinoma, and non-small-cell lung carcinoma [37].

GCS is involved in various malignant phenotypes, including cell growth, metastasis, and drug resistance [19,38]. The molecular mechanism of GCS on tumor progression and multi-drug resistance through the PI3K/AKT, MAPK/ERK, and c-Src/β-catenin signaling pathways is well-defined [39]. Additionally, GCS expression can be induced by anticancer drugs (viz., doxorubicin and cisplatin), leading to drug resistance in several types of cancer cells [40,41]. Therefore, cisplatin has been used in advanced CCA treatment alone or with other agents [9,42]. In the present study, cisplatin exhibited an anticancer effect in both the CCA cell lines in a dose- and time-dependent manner.

Interestingly, GCS and GBA2 expression were both significantly induced in the KKU-213A cell line at a lower dose of cisplatin (10 and 20 µM). The low GCS/GBA2 expression ratio highlighted the likelihood that aberrant ceramide glycosylation may be involved in the anticancer activity of cisplatin in CCA. However, the expression of GCS and GBA2 in KKU-100 were slightly changed following the cisplatin treatment. These results indicate that alteration of ceramide glycosylation upon cisplatin treatment may depend on each CCA cell line’s genetic background and malignant characteristics including anatomical location and malignant phenotypes. KKU-100 is a slow growing cell line that was established from a perihilar-CCA-containing wild type TP53 and mutant Kras, whereas KKU-213 is a highly aggressive CCA cell line which was established from intrahepatic CCA containing both TP53 and Kras mutations [24,25]. These findings agree with other studies on gemcitabine treatment and other anticancer agents, in which intracellular metabolic alterations and/or different mechanisms of drug resistance determine the differential response of each CCA cell line to particular chemotherapeutic agents [26,43,44,45].

Overexpression of GCS is mainly found in multi-drug-resistant cells in several types of cancer and contributes to a poor response to chemotherapy [31]. Inhibition of GCS shows increased chemosensitivity of cancer cells to various anticancer drugs (i.e., sorafenib, cisplatin, and doxorubicin) [42,46,47]. We further investigated whether GCS is implicated in CCA progression and drug resistance. Suppression of GCS using the chemical inhibitor (PPMP) or specific siRNA to GCS significantly reduced CCA cell growth and decreased the transcription levels of growth-related genes. Moreover, an anti-proliferative effect was seen in PPMP and GCS knockdown co-treatments. These results are consistent with previous studies in hepatocellular carcinoma and colon cancer, in which the disruption of ceramide homeostasis by GCS inhibition using RNA interference contributed to a reduction of tumor growth [22,48,49]. Moreover, GCS suppression using a chemical GCS inhibitor induced an increased ceramide level, leading to an inhibition of cell growth in melanoma cancer and head and neck cancer [22,40,50]. However, the effect of GCS on CCA cell growth is preliminary, and further study is needed on the association between GCS inhibition-induced ceramide production and CCA cell growth to confirm our findings.

Inhibition of GCS increased the chemosensitivity of CCA cell lines to low-dose cisplatin (10 and 20 µM), where GCS expression was induced. However, the decrease in the transcriptional levels of ABC transporter superfamily including ABCB1 (MDR1), ABCC1, ABCC3, and ABCG2 was not observed after GCS suppression (data not shown). This result indicates that induction of GCS expression by low-dose cisplatin may serve as a survival mechanism to protect ceramide levels from increased GBA2 activity and attenuate cisplatin-induced CCA cell death. Subsequently, inhibition of GCS (using PPMP) enhanced cisplatin-induced CCA apoptosis via up-regulation of cleaved caspase-3 and cleaved PARP1 and down-regulation of BCL-2. These findings are consistent with studies of head and neck cancer and colon cancer, in which GCS inhibition down-regulates MDR1 expression and induces ceramide-mediated apoptosis in cisplatin-resistant cells [41,48]. As such, GCS is a promising target to enhance the chemosensitivity of CCA to cisplatin. However, further study with other GCS inhibitors such as eliglustat, which is used for Gaucher’s disease in a clinical setting, and in vivo experiments are needed to clarify the significant role of GCS for chemoresistance in CCA. The accumulation of glycosphingolipids (GSLs), especially glucosylceramide, promotes cancer and drug resistance progression through various signal transduction mechanisms, including the c-Src/β-catenin, PI3K/AKT, and ERK pathways. The activation of both the AKT and ERK pathways plays a major role in the carcinogenesis of CCA and cisplatin resistance [51,52]. The present study demonstrates that phosphorylation and activation of ERK were increased following low-dose cisplatin treatment where inducible GCS expression was observed. Suppression of GCS by PPMP greatly reduced the phosphorylation and activation of ERK following cisplatin treatment, while no activation of AKT was observed after GCS inhibition. These findings suggest that GCS suppression enhanced cisplatin-induced CCA apoptosis through the attenuation of the ERK signaling pathway. These findings are consistent with published reports that GCS knockdown down-regulates anti-apoptotic protein BCL-2 by limiting the activation of the ERK signaling pathway [53].

In summary, our results demonstrate that GCS was up-regulated in CCA. The induction of GCS expression was detected in CCA cell lines following low-dose cisplatin treatment. Blocking ceramide glycosylation by inhibiting GCS attenuated the progression of CCA by reducing CCA cell growth and sensitizing CCA cells to low-dose cisplatin. Knockdown of GCS enhanced cisplatin-induced CCA apoptosis by diminishing the activation of the ERK-signaling pathway. As a result, targeting GCS may be a potential strategy for improving CCA treatment.

## Figures and Tables

**Table 1:** Association of GCS expression and clinicopathological features in CCA patients.

Clinicopathological Feature	Cases (n = 29)	GCS mRNA Expression Levels #		*p*-Value *
		Low	High	
**Sex**				
Male	15	10	5	0.474
Female	14	11	3	
**Age (years old)**				
≤50	5	4	1	0.677
>50	24	17	7	
**Tumor stage**				
I	1	1	0	0.690
II	4	3	1	
III	20	15	5	
IV	4	2	2	
**Normal stage (Lymph node)**				
0	17	13	4	0.561
I	12	8	4	
**Histological**				
Papillary carcinoma	10	9	1	0.124
Tubular adenocarcinoma	19	13	6	

# 1.5-fold used as cut-off value for dichotomizing low and high GCS expression groups. * *p*-value < 0.05 statistically significant based on chi-square test.

## Data Availability

The data presented in this study are included in this published article.

## Supplementary Materials

The following supporting information can be downloaded at: https://www.mdpi.com/article/10.3390/life12030351/s1, Figure S1: Original unedited blot of GCS and β-actin for representative Western blots used in Figure 4A of the manuscript; Figure S2: Original unedited blot of PARP, cleaved-PARP, caspase-3, cleavage-caspase-3, BCL-2, BAX, AKT, pAKT, ERK, pERK and β-actin for representative Western blots used in Figure 6A,B of the manuscript.
