# Supplementary material for: Inhibition of Ceramide Glycosylation Enhances Cisplatin Sensitivity in Cholangiocarcinoma by Limiting the Activation of the ERK Signaling Pathway
Source: Life (Basel). 2022 Feb 28;12(3):351. doi: 10.3390/life12030351 (PMC8949529; doi:10.3390/life12030351)

Figure S1. Original unedited blot of GCS and β-actin for representative Western blots used in Figure 4A of the manuscript.

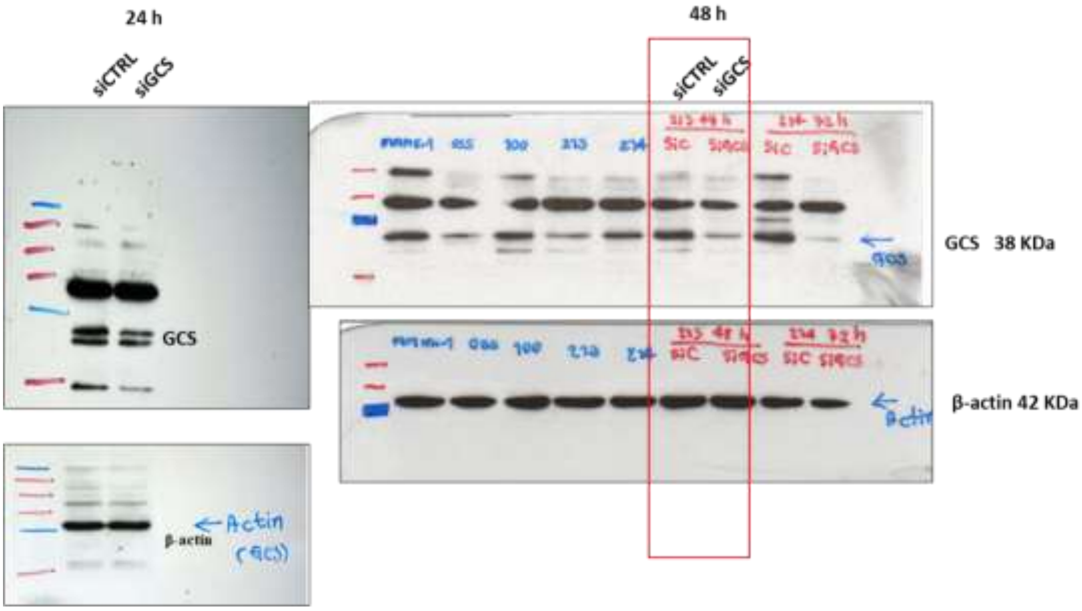

Figure S2. Original unedited blot of PARP, cleaved-PARP, caspase-3, cleavage-caspase-3, BCL-2, BAX, AKT, pAKT, ERK, pERK and β-actin for representative Western blots used in Figure 6A and 6B of the manuscript.

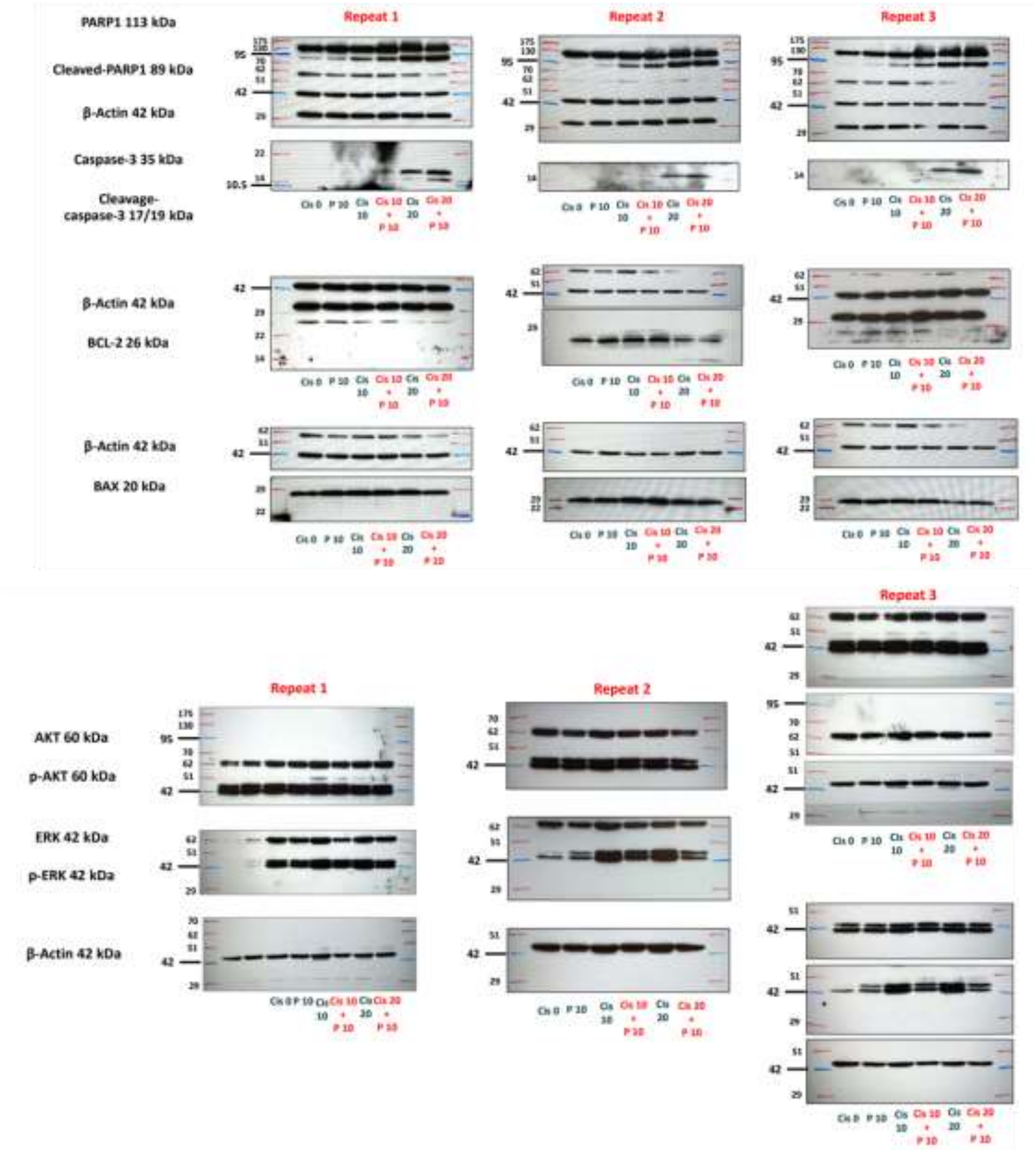

Supplement: Supplementary file 1 [file life-12-00351-s001.zip › life-1591310-supplementary.pdf]
